# Experimental Study on the Effect of Gradient Interface on the Mechanical Properties of Cu/WC_P_ Functionally Gradient Materials Using Digital Image Correlation Technique

**DOI:** 10.3390/ma15114004

**Published:** 2022-06-04

**Authors:** Hai Yu, Yunpeng Liu, Yunxiang Hu, Mingyang Ta

**Affiliations:** 1 School of Civil Engineering, North Minzu University, Yinchuan 750021, China; huyunxiang625@126.com (Y.H.); tmy_1258@126.com (M.T.); 2 School of Materials Science and Engineering, University of Science and Technology Beijing, Beijing 100083, China; jasonliu0514@163.com

**Keywords:** functionally gradient materials, digital image correlation, gradient interface, speckles, surface measurements, optical methods

## Abstract

In order to investigate the effect of gradient interface on the mechanical properties of Cu/WC_P_ functional gradient materials, digital image correlation technique was used to analyze the mechanical characteristics of laminated Cu/WC_P_ functional gradient material under tension load in the layer direction. In this paper, the deformation information of the specimens is obtained by the digital image correlation method. In order to obtain high-precision measurement results, speckle patterns with small spots and uniform distribution are prepared on the specimen surface by using small sample speckle preparation technology. The tensile experimental results showed that the incorporation of WC particles significantly improved the stiffness and strength of Cu/WC_P_ composites. Meanwhile, the plastic strain and plastic strain rate are non-uniform in each layer of the five-layer Cu/WC_P_ functional gradient material under the tension load along the layer direction. The plastic strain and plastic strain rate in each layer gradually increase along with the decreasing direction of WC content. It is found, from the analysis of experimental results, the existence of the gradient interface has an obvious inhibitory effect on the increase in plastic strain rate along the decreasing direction of WC content, and the specimen fracture location also has a certain relationship with the plastic strain rate, which reflects the important influence of the gradient interface on the mechanical properties of Cu/WC_P_ functional gradient materials.

## 1. Introduction

Functionally gradient materials (FGMs) are materials for which the composition and properties vary gradually as a function of position. Since their introduction by Kawasaki and Watanabe in high-temperature metal/ceramic aerospace components [1], FGMs have been used in a wide range of commercial applications, including aerospace, nuclear energy, wear-resistant coatings, biomedical equipment, nanotechnology, geological structures, high-temperature structures, and other fields [2,3]. FGMs are generally composed of two or more single materials that can be obtained through spatially consecutive changes according to specific rules [4]. According to the distribution of their components, functionally gradient materials can be classified into continuous gradient materials and laminated gradient materials [5,6,7,8]. Since the components of functionally gradient materials change with the position, functionally gradient materials generally have anisotropic mechanical properties. Therefore, the mechanical properties and mechanical behavior of functionally gradient materials have attracted the attention and research of a large number of scholars.

The relationship between microstructure and mechanical properties of Al2024/SiC functionally graded composites, prepared by powder metallurgy, was investigated by Erdemir et al. [2]. Their results suggested that the increase in microhardness and intermetallic formation play a major role in improving the mechanical properties of the composites. Tilbrook et al. [6] studied the influences on propagation trajectory for cracks initially oriented parallel to the layers in layered, graded alumina–epoxy composites, produced experimentally by infiltration of layered porous alumina bodies. Layered, graded material structures exhibit anisotropies in elastic properties and failure resistance that can strongly influence the propagation of cracks, and hence overall structural integrity. Uzun et al. [9] investigated the fatigue crack growth behavior in Al2124/SiC/10p FGMs, and found that FGMs exhibited good fatigue crack propagation resistance compared to traditional homogeneous composites. Xu et al. [10] studied the fatigue crack growth behavior of SiC particulates reinforced Al matrix graded composite, revealing that the crack growth rate decreased due to deflection and bifurcation at the interface. In addition, the difficulty and cost of manufacturing large-sized fracture specimens suitable for testing in experiments has led most researchers to conduct numerical computational analysis of functional gradient materials. Marur and Tippur [11] studied the fracture behavior of symmetrical three-point bending FGM beam samples through finite element and experiment analyses, and the results showed that when the crack was located at the interface between FGM substrate and uniform substrate, the fracture load of FGM sample was significantly higher than that of dual-material sample, and it was roughly the same as that of uniform sample. Gallego et al. [12] investigated the existing capabilities and limitations of numerical simulations of fracture problems in functional gradient materials using the finite element software ABAQUS. The importance of the numerical fit of the elastic properties in the FE model was analyzed in depth by means of a sensitivity study and a novel method was presented. The finite element method is studied for its use in cracked and uncracked plates made of functionally graded materials by Anlas et al. [13]. In this article, the material property variation was discretized by assigning different homogeneous elastic properties to each element. Finite element results were compared to existing analytical results and the effect of mesh size was discussed. The propagation of stress waves in functionally graded materials (FGMs) was studied numerically by means of the composite wave-propagation algorithm by Berezovski et al. [14].

Compared with the complex preparation process of continuous gradient materials, the metal-based functionally gradient composites with relatively simple preparation process [15] will be one of the development directions of laminated gradient materials. Metal-based functionally gradient composites have specific positional properties in components, in addition to performing their specific properties, metal-based functionally gradient composites are often required to withstand high stress or load in structures or components. For laminated functionally gradient materials, the change of material components is not uniform transition, but gradual transition layer by layer, which inevitably leads to the existence of interfaces between different components. The interface between layers plays an important role in the mechanical properties and even has an important influence on the damage and failure of structures or components under high load conditions. Metal-based functionally gradient composites are made of particle-reinforced metal-based composites with different volumes through gradient stacking and hot-pressing sintering according to the designed components. Due to the incorporation of particle reinforcement, the composites with different volume fractions exhibit different plasticity and toughness. After formation by gradient stacking hot-pressing sintering, the existence of gradient interface will inevitably lead to great changes in the overall mechanical properties. Therefore, it is a great significance to study the effect of gradient interface on the deformation and fracture mechanism of metal-based functionally gradient composites for the application of such materials under high load.

In order to study the effect of gradient interface on the mechanical properties of metal-based functionally gradient composites, five-layers Cu/WC_P_ functionally gradient materials were prepared by a powder metallurgy method. The deformation and mechanical behavior of multilayer Cu/WC_P_ FGMs under the tensile action along the layer direction was studied by digital image correlation method through experiments. The deformation characteristics of multilayer Cu/WC_P_ FGMs under tensile loading along the layer direction were analyzed.

## 2. Materials and Methods

### 2.1. Preparation of Functionally Gradient Materials

**Figure 1:**
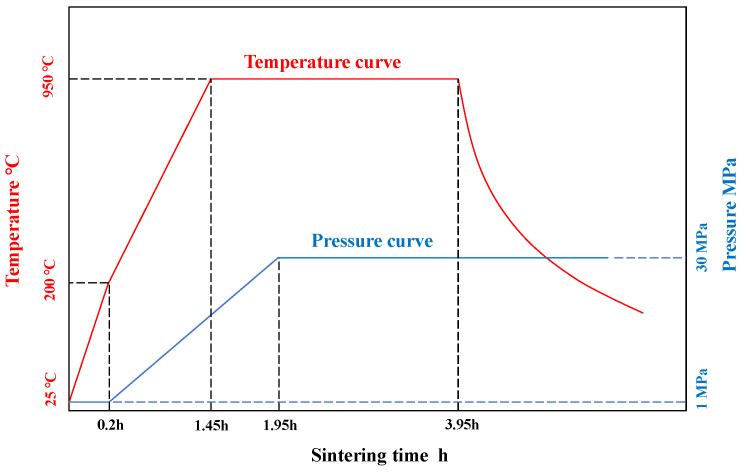
Sintering process of Cu/WC_P_ functionally gradient materials.

**Figure 2:**
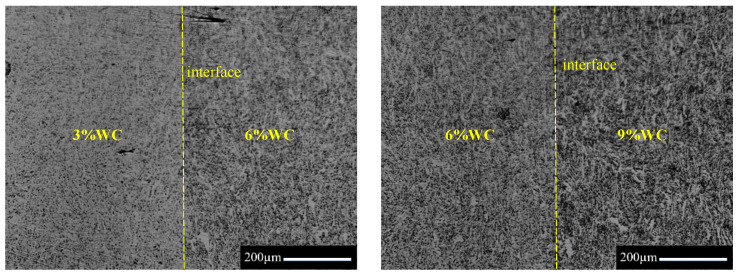

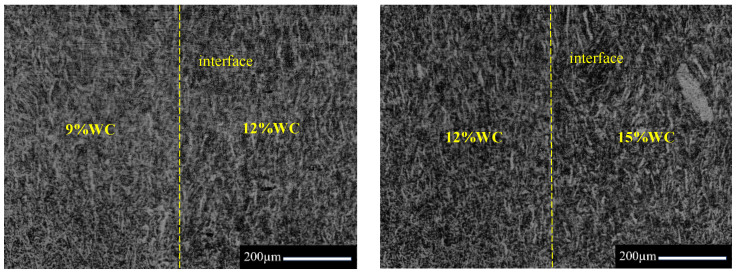
Optical images of Cu/WC_P_ composites with different WC contents.

In this paper, laminated Cu/WC_P_ functionally gradient materials were prepared by powder metallurgical process. The dendritic copper powder and irregular WC particles were used as raw materials (the average size of the WC particles used is 3.75 μm). In this study, five layers of Cu/WC_P_ functionally gradient materials were prepared by vacuum hot-pressing sintering, and the corresponding WC volume content of each layer was 3%, 6%, 9%, 12%, and 15%, respectively. The Cu/WC_P_ functionally gradient material is prepared by the laminated design method, the composite powder is laid in the membrane according to the preset order, and then hot-pressed sintering. The brief preparation processes were as follows: (1) powder mixing with a planetary ball mill; (2) pre-forming of the five layers uniformly mixed composite powder in a graphite mold; and (3) vacuum hot-pressing sintering of the pre-formed five-layer mixed powder at 950 °C. The specific sintering process is shown in Figure 1. Figure 2 shows the optical images of Cu/WC_P_ composites with different WC contents.

### 2.2. Digital Image Correlation Method

#### 2.2.1. Basic Principles of Digital Image Correlation

Digital image correlation (DIC) [16,17] is a non-contact optical technique that is capable of measuring full-field two-dimensional or three-dimensional surface deformations. Over the years, with the rapid advancement in computer vision technology, and with the availability of cheaper and more powerful digital image acquisition devices, DIC has gained increasing popularity among researchers in different fields [18,19]. Ahmed et al. [20] used DIC to investigate the fracture parameters and crack tip deformation field in continuous functionally graded (FG) glass-filled epoxy composite. Khazal et al. [21] calculated the fracture parameters, namely T-stress and stress intensity factors of a stepwise functionally graded material (FGM) by using the digital image correlation technique. Farouq et al. [22] investigated the mechanical properties and fracture analysis of a functionally graded material (FGM) made from sphere glass and epoxy resin composites using a hand lay-up process. The stress intensity factor is determined by the digital image correlation technique for the compact tension specimen. Abanto-Bueno et al. [23] studied the crack growth resistance behavior of functionally graded materials (FGMs) using the full-field measurement technique of digital image correlation.

DIC in fact is a subset of the research area of image registration. Essentially, DIC compares two digital images; one is the reference image corresponding to an undeformed state and the other is the deformed image. The DIC algorithm will search for the one-to-one correspondence of the points (pixels) in both images by matching the pixel intensities in an area with a unique image pattern. In order to obtain the displacements of each point of interest, a correlation criterion must be predefined to evaluate the similarity between the reference subset and the target subset. According to studies on the correlation criterion, the zero-mean normalized cross correlation (ZNCC) [24,25] criterion and the zero-mean normalized sum of squared differences (ZNSSD) [25,26] criterion were strongly recommended for practical use, since they are insensitive to the scale and offset changes of the target subset intensity. The equations are shown below:

(1) $$C_{ZNCC}(p)=\frac{\sum_{\Omega }\left[F(x,y)-Fm\right]\left[G(x_{t},y_{t})-Gm\right]}{\sqrt{\sum_{\Omega }{\left[F(x,y)-Fm\right]}^{2}}\sqrt{\sum_{\Omega }{\left[G(x_{t},y_{t})-Gm\right]}^{2}}}$$

(2) $$C_{ZNSSD}(p)={\sum_{\Omega }\left[\frac{F(x,y)-Fm}{\sqrt{\sum_{\Omega }{\left[F(x,y)-Fm\right]}^{2}}}-\frac{G(x_{t},y_{t})-Gm}{\sqrt{\sum_{\Omega }{\left[G(x_{t},y_{t})-Gm\right]}^{2}}}\right]}^{2}$$

where Ω is the selected subset of pixels. F(x,y) is the gray-level value at (x,y) in the reference image, and $G(x_{t},y_{t})$ is the gray-level value at $\left(x_{t},y_{t}\right)$ in the deformed image. Fm and Gm are the mean gray-level value of the subset in reference and deformed image, respectively. The deformation parameter p to be determined characterizes the shape function, or deformation mapping function, which relates the corresponding 2D image coordinates in the two images. The most widely used shape functions are first-order [16] and second-order [27].

(3) $$\{\begin{array}{c} x_{t}=x+u+u_{x}d_{x}+u_{y}d_{y}\\ y_{t}=y+v+v_{x}d_{x}+v_{y}d_{y} \end{array}$$

(4) $$\{\begin{array}{c} x_{t}=x+u+u_{x}d_{x}+u_{y}d_{y}+\frac{1}{2}u_{xx}d_{x}^{2}+u_{xy}d_{x}d_{y}+\frac{1}{2}u_{yy}d_{y}^{2}\\ y_{t}=y+v+v_{x}d_{x}+v_{y}d_{y}+\frac{1}{2}v_{xx}d_{x}^{2}+v_{xy}d_{x}d_{y}+\frac{1}{2}v_{yy}d_{y}^{2} \end{array}$$

where $d_{x}=x-x_{0}$, $d_{y}=y-y_{0}$ and the deformation parameter $p=(u,v,u_{x},u_{y},v_{x},v_{y})$. $\left(u_{xx},u_{xy},u_{yy},v_{xx},v_{xy},v_{yy}\right)$ are the additional second-order deformation parameters. For the detailed theory of DIC, such as correlation criterion, subset shape functions, nonlinear optimization, intensity interpolation, calculation path, speckle pattern quality, et al., please refer to [25,28,29,30,31,32].

During the past 40 years, dozens of DIC algorithms have been proposed; among which, the two most popularly used major categories of algorithms are subset-based local DIC [16,17] and finite element-based (FE-based) global DIC [33,34,35,36,37,38,39,40,41,42,43]. According to recent studies [44], which pointed out that when the subset (element) size is not very small and the local deformation within a subset (element) can be well approximated by the shape function used, the standard subset-based local DIC approach not only provides better results in measured displacements, but also demonstrates much higher computation efficiency. Therefore, we used subset-based local DIC to calculate the displacement on the surface of specimens under tensile loading in our experiment.

#### 2.2.2. Specimen Preparation and Experimental Set Up

In order to accurately obtain the deformation information of Cu/WC_P_ functionally gradient materials under tensile loads, a digital image correlation method was used in this paper. Since the strain on the specimen surface was calculated by a digital image correlation method, the surface of specimen needs to be covered with randomly distributed speckles. The speckle patterns quality of the specimen surface have a great influence on the measurement accuracy of the digital image correlation method. The speckle size and uniformity of speckles obtained by different speckle fabrication methods are very different. According to relevant literature [45], the ideal speckle size is 2~5 pixels. Generally, the artificial speckle patterns are produced by alternately spraying black and white paint on the surface of the specimen. In this paper, Cu/WC_P_ functionally gradient materials were prepared by vacuum hot-pressing sintering; due to the limitation of the mold size, the final formed Cu/WC_P_ functionally gradient material blanks were relatively small, leading to smaller specimens that could be tested after further processing. In order to solve the problem of preparing speckles for small specimens, white paint and carbon powder were used. First, the specimen surface was covered with a thin layer of white paint, then carbon powder was sprayed on the thin white paint, and the white spray was allowed to solidify and form a layer. The speckle patterns prepared in this way are fine and evenly distributed, which is suitable for speckle pattern preparation for small samples.

**Figure 3:**
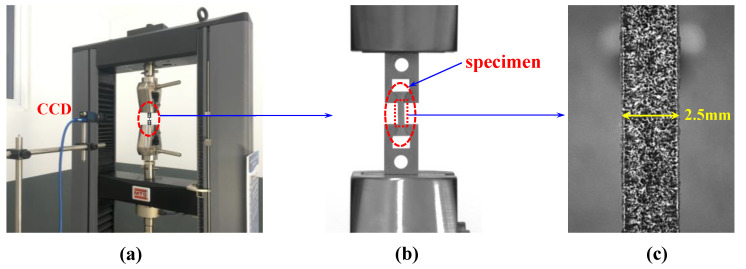
The experimental setup and speckle pattern on the surface of specimen: (**a**) the full experimental setup; (**b**) the chuck and specimen; (**c**) speckle pattern on the surface of specimen.

In this paper, the experiments were carried out on the material mechanical properties test system. Due to the influence of the size of the specimen, we designed and made a chuck that was suitable for the small sample in this experiment. In order to obtain high-quality speckle patterns, a camera with a long focal lens was used to record the speckle patterns of the specimen surface. The full experimental setup and details are shown in Figure 3.

## 3. Experimental Details

**Figure 4:**
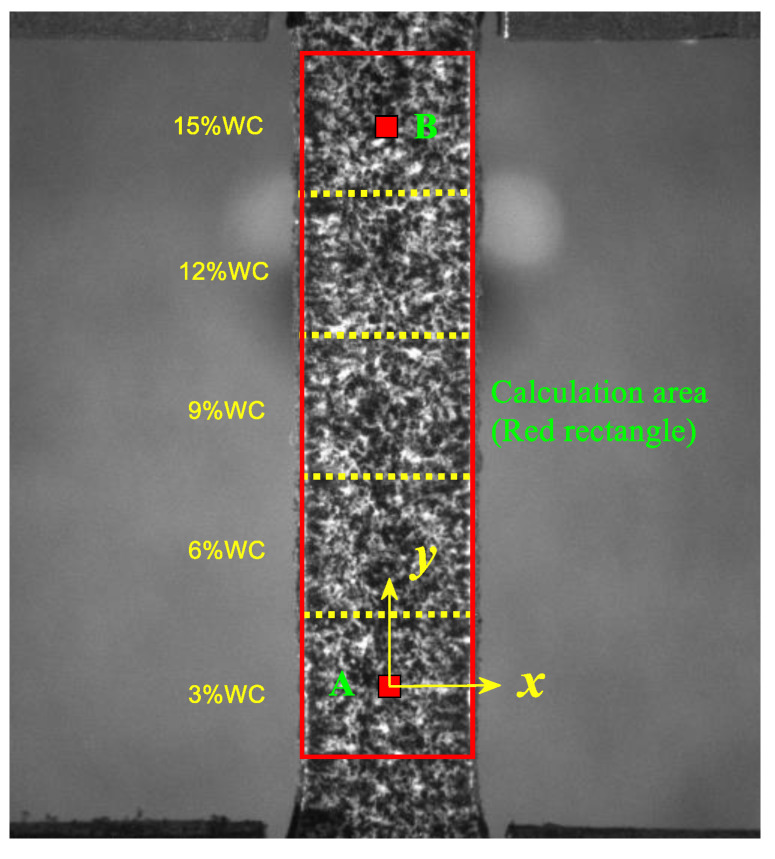
Schematic diagram of laminated Cu/WC_P_ functionally gradient material specimen and DIC calculation area (red rectangle).

First, tensile experiments on Cu/WC_P_ composites with different WC contents were conducted to obtain its mechanical parameters in a normal temperature environment, and standard dumbbell-shaped tensile samples were used as the tensile samples. Second, the mechanical properties of the five-layer Cu/WC_P_ laminated functionally gradient material were evaluated through tensile tests. Due to the limitations of the film size, the tensile test of the five-layer Cu/WC_P_ laminated functionally gradient material used a non-standard dumbbell-shaped specimen. The specimen properties were thickness H = 0.1 mm, width B = 2.5 mm, parallel segment length L = 10 mm, as shown in Figure 4. The red rectangular area in Figure 4 is the DIC calculation area. The length of each layer of the five-layer Cu/WC_P_ laminated functionally gradient material is 2 mm and the two marked points A and B are the central position in the first layer (3% WC) and the fifth layer (15% WC), respectively. The equipment used for the quasi-static loading in the experiment was the material mechanical properties test system (MTS E44.304). The loading frame is operated in displacement control mode, the loading rate is 0.5 mm/min, and the resolution of the image acquisition device is 2592 × 1944 pixels. During the whole loading process, the image acquisition equipment is used to record the speckle patterns on the surface of the specimen under different loads, step-by-step to calculate the strain of the specimen under the corresponding load.

## 4. Results and Discussion

### 4.1. Mechanical Properties of Cu/WC_P_ Composites

**Figure 5:**
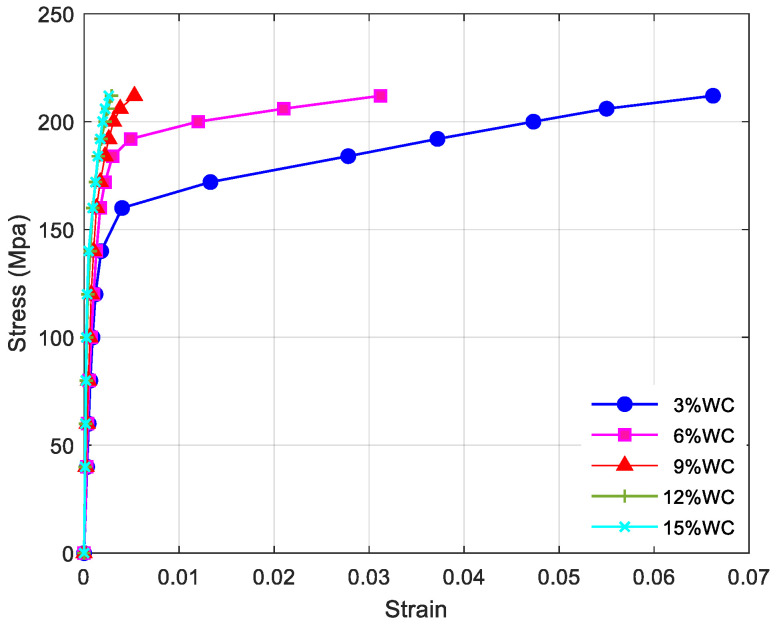
Stress–strain curves of Cu/WC_P_ composites under different volume content.

Table 1 shows the mechanical properties of Cu/WC_P_ composites with different volume content. It can be seen from Table 1 that the elastic modulus and tensile strength of Cu/WC_P_ composites both increase with an increase in the volume content of WC reinforced particles. The Poisson ratio gradually decreases with the increase in the volume content of WC-reinforced particles. The elastic modulus of Cu/WC_P_ composite with 15% WC volume content is 19.8% higher than that of Cu/WC_P_ composites with 3% WC volume content, and the tensile strength is increased by 40%. Therefore, the incorporation of WC particles makes the strength of Cu/WC_P_ composite increase significantly more than that of the stiffness. According to the theory of direct strengthening and indirect strengthening, with the increase of particle content, the direct strengthening effect on the copper matrix is increased, the tungsten carbide particles bear the stress transmitted by the copper matrix and reduce the stress on the copper matrix. Figure 5 shows the stress–strain curve of Cu/WC_P_ composites under different volume content. Since the digital image correlation method was used to calculate the strain of specimens under different loads in the experiment, the stress–strain curves obtained from the limited digital images recorded by CCD are discrete scatter plots. It can be seen from the Figure 5 that the Cu/WC_P_ composites under different volume content exhibit significantly different plastic properties. When the stress reached 214 MPa, the strains of Cu/WC_P_ composites with 3%, 6%, 9%, 12% and 15%WC volume content are 0.0662, 0.0312, 0.0053, 0.0029 and 0.0026, respectively. Due to the increase in the content of WC particles, the indirect strengthening effect of WC particles on the copper matrix has increased. More tungsten carbide particles refine the crystal grains of the copper matrix and increase the local dislocation density of the matrix. The slip of the metal matrix becomes difficult, so that the copper matrix has been strengthened, leading to a substantial decrease in the plastic deformation ability of the materials.

### 4.2. Effect of Gradient Interface on the Mechanical Properties of Cu/WC_P_ Functionally Gradient Materials

**Figure 6:**
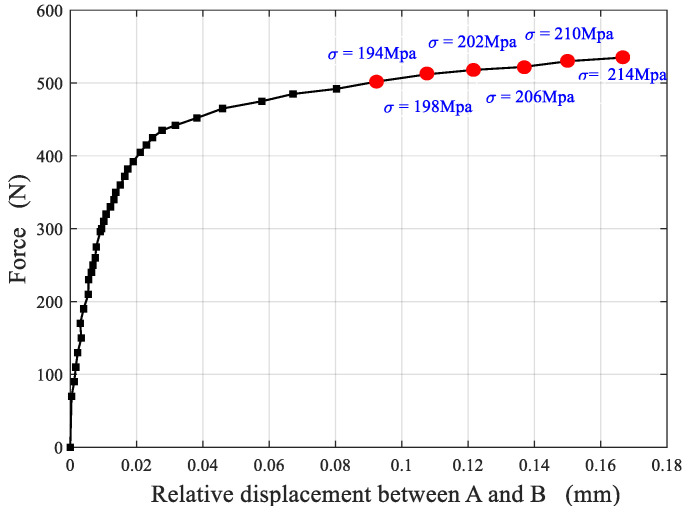
Force–displacement curve between point A and B of Cu/WC_P_ laminated functionally gradient material under tensile load.

**Figure 7:**
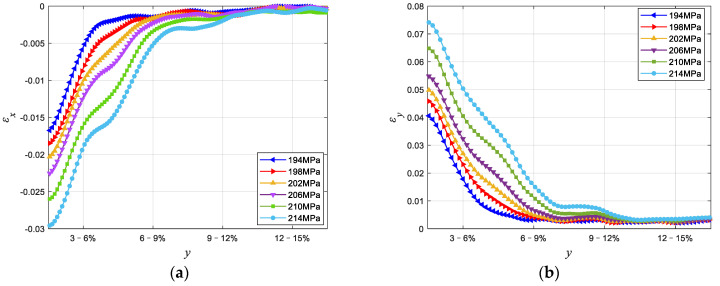
The strain curve for the y-coordinate between A and B on the axis of the specimen: (**a**) the transverse strain curves; (**b**) the longitudinal strain curves.

**Figure 8:**
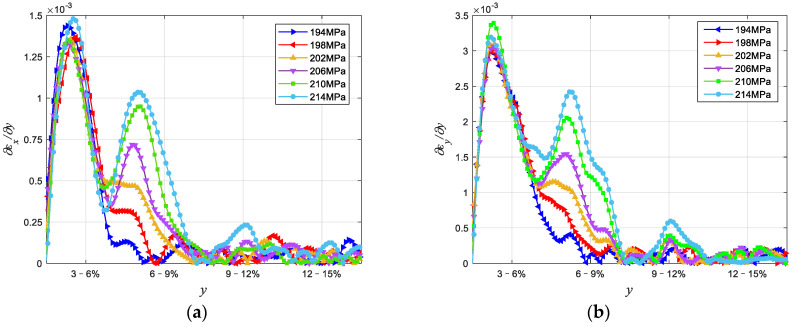
The strain rate curve for the y-coordinate between A and B on the axis of the specimen: (**a**) the transverse strain rate curves; (**b**) the longitudinal strain rate curves.

**Figure 9:**
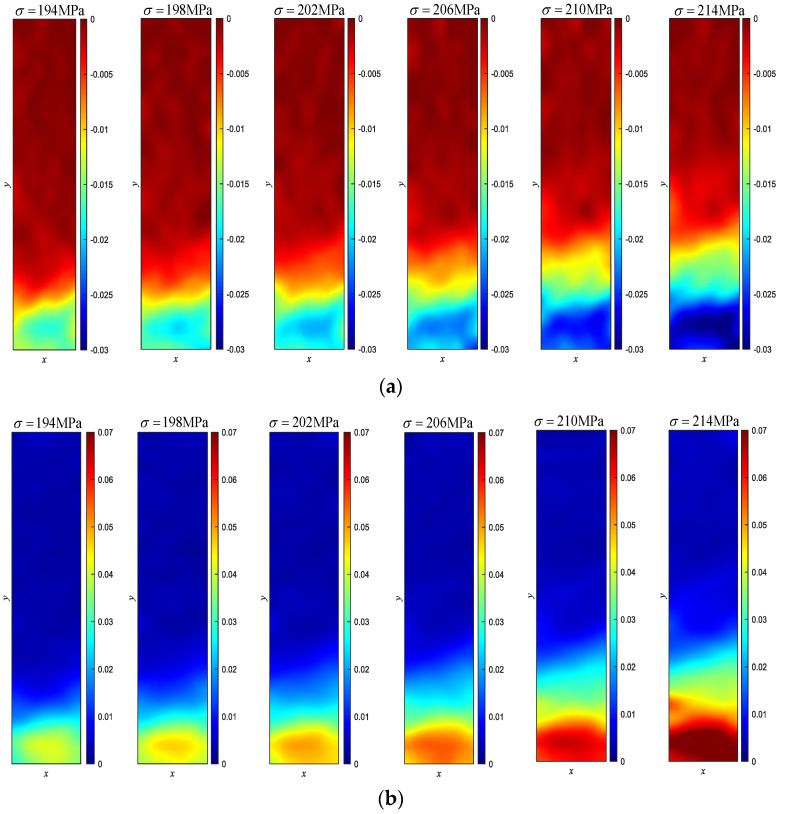
The distribution of strain on the specimen surface under different stress conditions: (**a**) the transverse strain; (**b**) the longitudinal strain.

Figure 6 is the force–displacement curve between point A and B of Cu/WC_P_ laminated functionally gradient material under tensile load. It can be seen from the experimental results and analysis in Section 4.1 that the inclusion of WC reinforced particles greatly changes the strength and plastic deformation capacity of the Cu/WC_P_ composites. Therefore, when the laminated Cu/WC_P_ FGMs are subjected to high stress or load in the structure or components, due to the lower strength and greater plastic deformation of Cu/WC_P_ with low WC content, this part of the material becomes the weak spots of the structure or component, and ultimately leads to the failure or destruction of the structure or component. In addition, for laminated Cu/WC_P_ FGMs, the existence of layer gradients or gradient interface will inevitably affect the overall mechanical properties. Through the tensile experiment studies, Figure 7 shows the strain curves of the y-coordinate between the two points A and B on the axis of the parallel segment of the sample under six different tensile stress states, where (a) is the longitudinal strain curve and (b) is the transverse strain curve. It can be seen from Figure 7 that all the layers have entered the plastic deformation stage for the six different tensile stress states. Due to the Cu/WC_P_ composite with high WC content has high tensile strength and low plastic deformation ability, the plastic deformation of the whole specimen mainly occurred in the area of WC content of 3%, 6%, and near the interface of 6–9%. Under the influence of layer gradient or gradient interface, the deformation in each layer along the layer direction is not uniform, but gradually increases along the decreasing direction of WC content. Judging from the entire strain curve, the curve is relatively smooth, and there is no fluctuation of strain curve at the gradient interface and nearby areas. Figure 8 shows the strain rate curves along the layer direction corresponding to the curves in Figure 7, where (a) is the longitudinal strain rate curve and (b) is the transverse strain rate curve, which reflects the variation of plastic strain along the layer direction. It can be seen that the overall plastic strain rate shows an increasing law under different tensile stresses along the layer direction of WC content decreases. Specifically, the obvious decrease of strain rate near the interface with WC content of 3–6% is caused by the influence of specimen ends. Near each interface, the increasing law of the plastic strain rate is disturbed to varying degrees because of the existence of gradient interface. As shown in Figure 8, the existence of gradient interface inhibits the increase of plastic strain rate near the gradient interface along the decreasing direction of WC content. In addition, combined with Figure 7 and Figure 8, there are both high plastic strain and plastic strain rate near the interface with WC content of 3% and 3–6%, making this region the weak area of the whole specimen. The position of maximum strain rate near the interface with WC content of 3–6% has a certain relationship with stress. With the increase in stress, the maximum point of strain rate gradually approaches the 3–6% interface from the side of WC content is 3%. When subjected to high stress or high load, this has greater impact on the damage and destruction of materials in the nearby area. Figure 9 is the distribution of strain on the specimen surface under different stress conditions, where (a) is the transverse strain and (b) is the longitudinal strain.

### 4.3. Fracture Location Analysis

**Figure 10:**
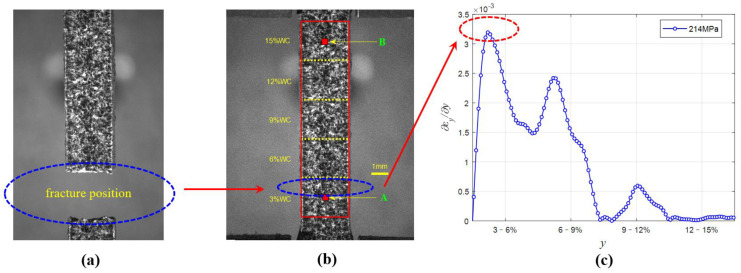
Schematic diagram of specimen fracture location. (**a**) Fracture morphology of specimen; (**b**) Fracture position of specimen; (**c**) Strain rate curve in y direction when the stress is 214 MPa.

Figure 10 is the schematic diagram of the fracture position of the five-layer tensile sample. It is obvious that the fracture of the sample is relatively smooth and there is relatively obvious plastic deformation near the fracture. From the perspective of fracture location, the fracture occurred in the layer with 3% WC content and was close to the area near the 3–6% interface, which precisely corresponded to the extreme point of strain rate. On the one hand, the fracture occurred at this position because of the influence of the end effect. Due to the existence of the dumbbell end, the plastic strain near the end will be significantly reduced. On the other hand, it can be seen from Figure 7 and Figure 10 that the fracture position does not occur near the maximum plastic deformation, but at the extreme point of the plastic strain rate, indicating that the plastic strain rate has a certain effect on the damage and failure of the Cu/WC_P_ functionally gradient material. It also reflects the influence of plastic strain rate on the mechanical properties of Cu/WC_P_ functionally gradient materials.

## 5. Conclusions

In this work, digital image correlation technique was used to investigate the effects of gradient interface on the mechanical properties of Cu/WC_P_ functional gradient materials. The following main conclusions can be drawn from this study:

The elastic modulus and tensile strength of Cu/WC_P_ composites prepared by powder metallurgy were improved due to the incorporation of WC reinforcement particles; especially, the tensile strength was improved more significantly. The elastic modulus of Cu/WC_P_ composites with 15% WC volume increases by 19.8% and the tensile strength increases by 40% compared with that of Cu/WC_P_ composites with 3% WC volume. 

In terms of plastic deformation capacity, the incorporation of WC reinforcement particles significantly improves the stiffness and strength of Cu/WC_P_ composites, but its plastic deformation capacity significantly and substantially decreases. In the single-layer tensile experiment, when the maximum stress is 214 MPa, the strain of Cu/WC_P_ composites with 3% WC volume fraction is 2.12, 10.51, 22.83, and 25.45 times higher that of Cu/WC_P_ composites with 6%, 9%, 12%, and 15% volume, respectively. 

The tensile experiment results of five-layer Cu/WC_P_ FGMs show that the plastic deformation in each layer is non-uniform along the direction of layer, but gradually increases along the direction of WC content reduction. The strain curves do not show fluctuations at the gradient interface and nearby areas, but due to the existence of gradient interface, the increasing law of strain rate at the gradient interface and its vicinity is disturbed to different degrees.

From the overall results, the existence of the gradient interface significantly inhibits the increase in plastic strain rate in the decreasing direction of WC content near the gradient interface. In addition, from the fracture position of the specimens, the fracture position occurs at the maximum value of strain rate in y direction, and the maximum value is 0.0034. The plastic strain rate has a certain influence on the damage and failure of Cu/WC_P_ functionally gradient materials, which reflects the influence of the plastic strain rate on the mechanical properties of Cu/WC_P_ functionally graded materials. In future work, we will study the influence of the gradient interface on the damage of Cu/WC_P_ functionally gradient materials from a microscopic perspective.

## Figures and Tables

**Table 1:** Mechanical properties of Cu/WC_P_ composites with different volume content.

WC Content	Poisson Ratio	Elastic Modulus (GPa)	Tensile Strength (MPa)
3%	0.36	116	219
6%	0.345	121	242
9%	0.335	127	264
12%	0.327	133	289
15%	0.32	139	307

## Data Availability

Not applicable.

## Abbreviations

The following abbreviations are used in this manuscript:

DIC	Digital Image Correlation
FGMs	Functionally Gradient Materials
